# Quantum Advantages of Communication Complexity from Bell Nonlocality

**DOI:** 10.3390/e23060744

**Published:** 2021-06-13

**Authors:** Zhih-Ahn Jia, Lu Wei, Yu-Chun Wu, Guang-Can Guo

**Affiliations:** 1 CAS Key Laboratory of Quantum Information, School of Physical Sciences, University of Science and Technology of China, Hefei 230026, China; wuyuchun@ustc.edu.cn (Y.-C.W.); gcguo@ustc.edu.cn (G.-C.G.); 2 CAS Center For Excellence in Quantum Information and Quantum Physics, University of Science and Technology of China, Hefei 230026, China; 3 School of the Gifted Young, University of Science and Technology of China, Hefei 230026, China; cox@mail.ustc.edu.cn

**Keywords:** Bell nonlocality, entanglement, communication complexity

## Abstract

Communication games are crucial tools for investigating the limitations of physical theories. The communication complexity (CC) problem is a typical example, for which several distributed parties attempt to jointly calculate a given function with limited classical communications. In this work, we present a method to construct CC problems from Bell tests in a graph-theoretic way. Starting from an experimental compatibility graph and the corresponding Bell test function, a target function that encodes the information of each edge can be constructed; then, using this target function, we can construct a CC function, and by pre-sharing entangled states, its success probability exceeds that of the arbitrary classical strategy. The non-signaling protocol based on the Popescu–Rohrlich box is also discussed, and the success probability in this case reaches one.

## 1. Introduction

Bell nonlocality [1,2,3,4] is one of the most distinctive features that distinguish quantum mechanics from classical mechanics. It is an experimentally verified phenomenon and now serves as a crucial resource for many quantum information tasks, such as quantum computation [5], quantum key distribution (QKD) [6], quantum random number generation [7], and the communication complexity (CC) problem [8]. Among these tasks, CC problems for which distributed parties jointly calculate a function with limited communications are of great importance for investigating the limitations of different physical theories [4,8]. For instance, the set of calculable functions and the success probabilities for calculating a given function may be different for local hidden variable (LHV) theory [2], quantum theory, and non-signaling theory.

CC problems, originally introduced by Yao [9], concern the following question: what is the minimal amount of communication necessary for two or more parties to jointly calculate a given multivariate function $f(x_{1},\cdots ,x_{n})$ where the *k*-th party only knows his own input $x_{k}$ but no information about the inputs of other parties initially? It has been shown from different perspectives that entanglement and Bell nonlocality are closely related to the quantum advantage of the CC problem; see Refs. [8,10,11,12,13,14,15]. Violation of Bell inequalities often leads to quantum advantages of CC problems [8,10,11,12,13] and it is also argued that the quantum advantage of CC problem implies violation of Bell inequalities [14,15]. However, many of the results above are existence proof. In practice, to utilize Bell nonlocality to obtain quantum advantages of a real CC problem, one needs to consider how to explicitly translate the Bell test into a CC problem. In this work, we systematically explore the translation of a Bell test into CC problem via the graph-theoretic method.

We are mainly interested in the CC problems for which only limited classical communications are allowed and the goal for each party is to calculate the function with as high success probability as possible. By introducing the concept of the experimental compatibility graph and its corresponding Bell test function, we explore the relationship between the Bell nonlocality and quantum advantage of CC problems. We show that, from an arbitrary experimental compatibility graph $G^{e}$, we can construct a corresponding CC problem $F_{G^{e}}$ for which the quantum protocol exhibits a success probability that exceeds the that of all classical protocols. We also investigate the possibility of using a non-signaling box to solve CC problems, and we show that it has an advantage over all quantum protocols.

The paper is organized as follows: in Section 2, we introduce several graph-theoretic concepts related to Bell nonlocality, including the experimental compatibility graph, compatibility graph, and Bell test functions; in Section 3, we provide the basics of CC problems and define the quantum advantages of the protocol; in Section 4, we present a class of functions based on an arbitrary given experimental graph $G^{e}$ for which quantum protocols exhibit advantages; finally, in the last section, we make some concluding remarks.

## 2. Bell Inequalities from Compatibility Graphs

**Figure 1:**
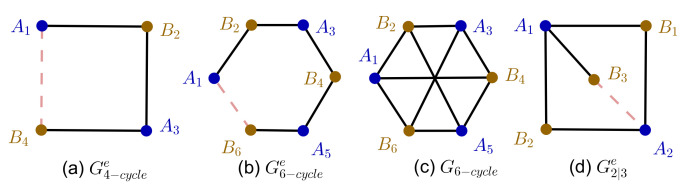
Depiction of the experimental compatibility graph and measurement compatibility graph. (**a**) Experimental compatibility graph $G_{4-cycle}^{e}$ of CHSH inequality, which is a 4-cycle graph; (**b**) experimental compatibility graph $G_{6-cycle}^{e}$ of 6-cycle Bell inequality; (**c**) measurement compatibility graph $G_{6}$ corresponding to $G_{6-cycle}^{e}$; (**d**) non-cycle experimental compatibility graph $G_{2|3}^{e}$.

Let us now introduce a general framework for *n*-party Bell inequalities based on a set of *n*-point correlation functions $E\left(x_{i_{1}},x_{i_{2}},\cdots ,x_{i_{n}}\right)=\langle x_{i_{1}}⊗x_{i_{2}}⊗\cdots ⊗x_{i_{n}}\rangle$. Many pertinent classes of Bell inequalities are of this correlator form; see, e.g., Refs. [4,16]. To start with, let us first introduce a useful mathematical tool, compatibility graphs. For a set of measurements $M=\{M_{1},\cdots ,M_{n}\}$, we can assign a corresponding graph $G_{M}$ called the *measurement compatibility graph* [17], whose vertices are labeled by measurements, and there is an edge between two vertices if the corresponding measurements are compatible; i.e., they can be measured simultaneously. We denote the vertex set of the graph *G* as V(G) and the edge set as E(G), and an edge is a pair $\langle ij\rangle :=\left(M_{i},M_{j}\right)\in V\left(G\right)\times V\left(G\right)$. Similarly, we can introduce the *experimental compatibility graph and hypergraph* $G_{M}^{e}$ [17], in which the vertices are labeled with the measurements involved in the experiment, and an edge represents two or more jointly measured measurements in the experiment. For two-party case, each edge consists of two measurements, and $G_{M}^{e}$ is a subgraph of $G_{M}$, while for the *n*-party (n>2) case, each edge consists of *n* vertices; thus, $G_{M}^{e}$ is a hypergraph. See Figure 1b,c for an illustration of the compatibility graph and two-party experimental compatibility graph.

In a typical *n*-party Bell scenario, the experimenters share an *n*-partite system. According to an experimental compatibility graph $G^{e}$, they can choose a set of measurements $x_{i_{1}},x_{i_{2}},\cdots ,x_{i_{n}}$ for joint measurements, where $x_{i_{k}}$ is the measurement chosen by the *k*-th party. After many runs of experiments, they obtain a set of *n*-point correlation functions $\left\{E\left(x_{i_{1}},x_{i_{2}},\cdots ,x_{i_{n}}\right)|\langle i_{1}i_{2}\cdots i_{n}\rangle \in E\left(G^{e}\right)\right\}$. To determine whether the obtained measurement statistics are local, viz., obey the LHV theory or not, a function must be calculated,

(1) $$\begin{array}{cc} B_{G^{e}}= & \sum_{\langle i_{1}i_{2}\cdots i_{n}\rangle \in E\left(G^{e}\right)}\gamma_{\langle i_{1}i_{2}\cdots i_{n}\rangle }E\left(x_{i_{1}},x_{i_{2}},\cdots ,x_{i_{n}}\right)\\ \; & +\sum_{\langle i_{1}i_{2}\cdots i_{n-1}\rangle \in E\left(G^{e}\right)}\gamma_{\langle i_{1}i_{2}\cdots i_{n-1}\rangle }E\left(x_{i_{1}},x_{i_{2}},\cdots ,x_{i_{n-1}}\right)\\ \; & +\cdots +\sum_{x_{i}\in V\left(G\right)}\gamma_{\langle x_{i}\rangle }E\left(x_{i}\right), \end{array}$$

which we refer to as the *Bell test function*. In this work, we mainly focus on the homogenous case; that is, for the *n*-party Bell test, the Bell test function only contains *n*-point correlation functions. It is also convenient for our purpose to assume that $\gamma_{\langle i_{1}i_{2}\cdots i_{n}\rangle }=\pm 1$. In this case, different colors of edges of $G^{e}$ represent different coefficients; if $\gamma_{\langle i_{1}i_{2}\cdots i_{n}\rangle }=+1$, the edge is drawn as a black solid line and deemed a positive edge, and if $\gamma_{\langle i_{1}i_{2}\cdots i_{n}\rangle }=-1$, the edge is drawn as a red dashed line and deemed a negative edge, as depicted in Figure 1.

Note that for an *n*-party Bell experiment, $G^{e}$ is usually an *n*-partite graph, as the measurements of each party are usually chosen as incompatible measurements. In an LHV world, the value of the test function lies in the range $R_{LHV}=\left[B_{C}^{1},B_{C}^{2}\right]$; e.g., for the Clauser–Horne–Shimony–Holt (CHSH) type of Bell test function $B_{CHSH}$, the range is $R_{LHV}=\left[-2,2\right]$ [16]. However, for quantum theory, the value may lie outside the LHV range $R_{LHV}$; this is called the quantum violation of Bell inequality, which means that quantum theory is not consistent with the LHV assumption. Similar to LHV theory, there also exists a quantum range $R_{Q}=\left[B_{Q}^{1},B_{Q}^{2}\right]$ of the value of the Bell test function; e.g., for CHSH type of Bell test function, it is $R_{Q}=\left[-2\sqrt{2},2\sqrt{2}\right]$, and this kind of quantum bound is known as Tsirelson bound. Is it possible for a Bell test function to violate the quantum range? The answer is yes; there are many different kinds of approaches to understand quantum theory from outside; e.g., in non-signaling theory [18], the Bell test function may reach its functional minimal and maximal values. To summarize, we have the following Bell inequalities for a given experiment compatibility graph:

(2) $$B_{G^{e}}\overset{LHV}{\in }R_{LHV}\overset{Q}{\subseteq }R_{Q}\overset{NS}{\subseteq }R_{NS}.$$

Note that for a given experimental compatibility graph, the Bell test function is, in general, not unique.

Another crucial issue regards the kind of experimental compatibility graph that can be used to test Bell nonlocality. A necessary condition for this is the following [17,19]: the compatibility graph corresponding to $G^{e}$ is non-chordal. Chordal graphs are those that do not have any induced cycle with a size of more than three. From Vorob’yev theorem [20,21], if the compatibility graph *G* corresponding to $G^{e}$ is chordal, then there always exists a global joint probability distribution that can reproduce all marginal probability distributions that we obtained from the experiment. A result of Fine [22] further suggests that the existence of this kind of global joint probability distribution is equivalent to the existence of an LHV model for all involved measurements. Thus, for the chordal graph, the measurement statistics are always reproducible by the LHV model. In a recent work [23], it was claimed that the above condition is also a sufficient condition.

Here, we present two examples for convenience of our later discussions. We recommend readers to read Refs. [17,19,23,24,25] for more examples.

### 2.1. Example 1

The first example is 2m-cycle Bell inequality. The experimental compatibility graph is a 2m-cycle, for which $A_{1},A_{3},\cdots ,A_{2m-1}$ are observables chosen by Alice and $B_{2},B_{4},\cdots ,B_{2m}$ are observables chosen by Bob; the i-th vertex connects with the (i+1)-th vertex. The Bell test function is thus

(3) $$\begin{array}{cc} B_{G_{\mathrm{cycle}}^{e}}= & \sum_{i=1,3,\cdots ,2m-1}\gamma_{i}E\left(A_{i},B_{i+1}\right)+\sum_{i=2,4,\cdots ,2m}\gamma_{i}E\left(A_{i+1},B_{i}\right) \end{array}$$

note that, here, $\gamma_{i}=\pm 1$, and the number of $\gamma_{i}=-1$ must be odd to ensure that it can test Bell nonlocality. As proved in [26,27], Bell inequality is

(4) $$|B_{G_{\mathrm{cycle}}^{e}}|\overset{LHV}{\leq }2m-2\overset{Q}{\leq }2m\cos\frac{\pi }{2m}\overset{NS}{\leq }2m.$$

When m=2, the experimental compatibility graph is a 4-cycle graph, as depicted in Figure 1a; the corresponding Bell inequality is the CHSH inequality.

It is worth mentioning that, although we can construct a Bell test from the arbitrary non-chordal graph, the LHV bound (which corresponds to independent number calculation of a graph) and non-signaling boundary can easily be obtained, but the maximum quantum violation (which corresponds to the Lovász number calculation of a graph [24,28]) is usually very difficult to calculate. The example corresponding to non-cycle experimental compatibility graph can also be constructed.

### 2.2. Example 2

The experimental compatibility graph of this Bell test is shown in Figure 1d. We denote the graph as $G_{2|3}^{e}$, and the subscripts here is used to indicate that Alice chooses two observables and Bob chooses three observables to measure. The corresponding Bell test function is

(5) $$\begin{array}{cc} B_{G_{2|3}^{e}}= & E\left(A_{1},B_{1}\right)+E\left(A_{2},B_{1}\right)+E\left(A_{2},B_{2}\right)+E\left(A_{1},B_{2}\right)\\ \; & +E\left(A_{1},B_{3}\right)-E\left(A_{2},B_{3}\right). \end{array}$$

The LHV bound is 4, and the non-signaling bound is 6, but the exact quantum bound remains unknown.

## 3. Communication Complexity Problems

Now, let us recall the formal definition of communication complexity; for further information, we refer the reader to Refs. [29,30,31]. For simplicity, consider the two-party case, for which Alice and Bob try to calculate a bivariate function $f:B^{n}\times B^{n}\to B$ collaboratively, where B denotes the binary set {0,1} or {±1}. An *r*-round communication complexity protocol P for computing function f(x,y) is a distributed algorithm consisting of a set of *r* functions $f_{1},\cdots ,f_{r}:\cup_{m\geq 0}B^{m}\to \cup_{m\geq 0}B^{m}$. Alice first individually calculates function $f_{1}\left(x\right)=v_{1}$ and sends the result to Bob; after Bob receives the result, he calculates function $f_{2}\left(y,v_{1}\right)=v_{2}$ and sends the result to Alice, etc. Each act of communication is called a round. We suggest that the protocol P is valid for calculating f(x,y) if the last message sent (i.e., $v_{r}=f_{r}\left(x,v_{1},\cdots ,v_{r-1}\right)$ by Alice or $v_{r}=f_{r}\left(y,v_{1},\cdots ,v_{r-1}\right)$ by Bob) equals f(x,y) for all possible input values of x,y. The communication complexity of the protocol P is then defined as the $C_{P}\left(f\right)=|v_{1}\left|+\cdots +\right|v_{r}|$, where $|v_{i}|$ denotes the number of bits of the message $v_{i}$. The protocol defined above is deterministic. For the bounded-error case, Alice and Bob can toss coins individually or jointly to choose the input at each round, and the protocol P has to calculate *f* with a success probability greater than or equal to a fixed value 1−δ, where δ is usually chosen as 1/3, viz, $P_{succ}\geq 2/3$. We assume that if Bob guesses the value f(x,y) as z=0,1 during the final round, the successful probability will be

(6) $$p_{succ}\left(P\right)=\sum_{x,y}p\left(x,y\right)p\left(z=f\left(x,y\right)|x,y\right).$$

The bounded-error communication complexity is denoted as $C_{P}^{be}\left(f\right)$, which is the number of communicated bits in the protocol such that $p_{succ}\geq 1-\delta$ for some δ<1/2.

The bounded-error communication complexity problem concerns the problem of obtaining the lower bound of the amount of communication needed for all parties to obtain the value of a given function *f* with successful probability $P_{succ}\geq 1-\delta$. We can naturally ask the inverse question: what is the highest successful probability for calculating the function *f* if the amount of communication C(f) is restricted to be upper bounded $C\left(f\right)\leq C_{bd}$ ? Note that unlike in the regular communication complexity problem where the bound of successful probability 1−δ does not matter so much, in this kind of CC problem, the communication bound is of considerable importance. Since there exists a trivial protocol for calculating the arbitrary function *f*, for which Alice communicates her entire input to Bob, $p_{succ}$ can always reach 1 if the allowed communication is greater than or equal to min{|x|+1,|y|+1}.

There are two types of quantum communication complexity protocols: (i) the preparation measurement protocol and (ii) the entanglement-assisted protocol, which is similar to the categorification of quantum key distribution protocol. In this work, we mainly discuss the entanglement-assisted protocol.

The performance of a usual CC protocol P is characterized by the amount of communication, i.e., classical or quantum bits $C(P,f|p_{\mathrm{succ}})$ required to achieve the success probability $p_{\mathrm{succ}}$. The quantum advantage of the CC problem is that there exists a quantum protocol $P_{Q}$ such that for any classical protocol $P_{C}$, we have $C\left(P_{C},f|p_{\mathrm{succ}}\right)>C\left(P_{Q},f|p_{\mathrm{succ}}\right)$.

The performance of the CC protocol P for calculating function *f* can also be characterized by the maximal achievable success probability $p_{\mathrm{succ}}\left(P,f|C_{bd}\right)$ given a bounded amount of communication $C_{bd}$. Here, the communication could be classical bits or qubits; we suggest that there is a quantum advantage for the ICC problem for calculating *f* if there is a quantum protocol $P_{Q}$ such that $p_{\mathrm{succ}}\left(P_{Q},f|C_{bd}\right)>p_{\mathrm{succ}}\left(P_{C},f|C_{bd}\right)$ for all classical protocol $P_{C}$.

There is a simple and well-known example of the CC problem in Ref. [32], for which Alice and Bob receive bit strings $\left(x,a\right)\in B^{2}$ and $\left(y,b\right)\in B^{2}$, respectively, and they tend to calculate a function *f* given by the following language:

(7) $$L_{\mathrm{Bell}}=\left\{\left(x,a;y,b\right)\in B^{2}\times B^{2}|a⊕b=x\wedge y\right\}.$$

All input strings distribute uniformly, and the two parties are allowed to exchange only two classical bits. Their goal is to calculate $L_{\mathrm{Bell}}$ with as high successful probability as possible. In Ref. [10], Brukner et al. present the optimal classical protocol and prove that by using entangled quantum states that violate CHSH inequality, the quantum solution of the problem has a higher success probability than that of the optimal classical protocol, thus exhibiting the quantum advantage. This protocol works in the entanglement-assisted sense.

## 4. From Bell Inequality Violation to the Quantum Advantage for ICC Problems

We now discuss how to translate a Bell test into an ICC problem using a compatibility graph. To start with, let us consider the two-party case. For a given experimental compatibility graph $G^{e}$, which is a bipartite graph, the vertices are labeled with $x_{A}=v_{1},\cdots ,v_{n}$ by Alice and $x_{B}=u_{1},\cdots ,u_{m}$ by Bob. There are some edges corresponding to $\gamma_{\langle ij\rangle }=1$ (called positive edges, drawn as black solid edge in Figure 1) and some others corresponding to $\gamma_{\langle ij\rangle }=-1$ (called negative edges, drawn as red dashed edge in Figure 1). We introduce a function that we refer to as the target function

(8) $$t\left(x_{A},x_{B}\right)=\left\{\begin{array}{c} 0,\;\mathrm{for}\;\;\langle v_{i}u_{j}\rangle \;\;\mathrm{positive}\;\mathrm{edge},\\ 1,\;\mathrm{for}\;\;\langle v_{i}u_{j}\rangle \;\;\mathrm{negative}\;\mathrm{edge}. \end{array}\right.$$

Consider the following two-party scenario: Alice and Bob receive $\left(x_{A},y_{A}\right)$ and $\left(x_{B},y_{B}\right)$, respectively, where $y_{A},y_{B}=\pm 1$ and $x_{A}=1,\cdots ,n$, $x_{B}=1,\cdots ,m$, and the condition $\langle x_{A}x_{B}\rangle \in E\left(G^{e}\right)$ (i.e. it is an edge of the experimental compatibility graph $G^{e}$) are promised. The function they calculate is

(9) $$F_{G^{e}}\left(x_{A},y_{A};x_{B},y_{B}\right)=y_{A}y_{B}{\left(-1\right)}^{t(x_{A},x_{B})}.$$

Note that this is a partial function; for some inputs, the function is not defined—see Table 1 for an example. In this way, we can construct a CC function from an arbitrary given experimental compatibility graph.

For the *n*-party case, the corresponding experimental graph is an *n*-partite hypergraph; the vertices of *k*-th party are labeled with $x_{k}=u_{1}^{k},\cdots ,u_{m_{k}}^{k}$; the edge $\langle u_{i_{1}}^{1}\cdots u_{i_{n}}^{n}\rangle$ consists of *n* vertices, one from each party. Similar to the two-party case, we can define the target function

(10) $$t\left(x_{1},\cdots ,x_{n}\right)=\left\{\begin{array}{c} 0,\;\mathrm{for}\;\;\langle u_{i_{1}}^{1}\cdots u_{i_{n}}^{n}\rangle \;\;\mathrm{positive}\;\mathrm{edge},\\ 1,\;\mathrm{for}\;\;\langle u_{i_{1}}^{1}\cdots u_{i_{n}}^{n}\rangle \;\;\mathrm{negative}\;\mathrm{edge}. \end{array}\right.$$

The function to be calculated is

(11) $$F_{G^{e}}\left(x_{1},y_{A};\cdots ;x_{n},y_{n}\right)=y_{1}\cdots y_{n}{\left(-1\right)}^{t(x_{1},\cdots ,x_{n})}.$$

The CC problem to be solved is as follows: the *n* parties try to calculate the function (11), and the *k*-th party receives the bit string $\left(x_{k},y_{k}\right)$ with $x_{k}=u_{1}^{k},\cdots ,u_{m_{k}}^{k}$. The probability distribution for the input strings is

(12) $$p\left(x_{1},y_{1};\cdots ;x_{n},y_{n}\right)=\frac{1}{2^{n}}\times \frac{1}{\left|E(G^{e})\right|}.$$

Each party is allowed to broadcast one classical bit of information, and *n* parties broadcast the information simultaneously such that their broadcast bits are independent.

### 4.1. Optimal Classical Protocol

Let us now introduce an optimal classical protocol $P_{C}$ for the above CC problem. To make things clearer, we take the two-party case as an example. The main step is to calculate the target function part ${\left(-1\right)}^{t(x_{A},x_{B})}$. To do this, Alice and Bob firstly relabel their vertices as $x_{A}^{\prime }$ and $x_{B}^{\prime }$ such that the values $x_{A}^{\prime }+x_{B}^{\prime }$ are different for different edges. This can be achieved since $G^{e}$ is a finite graph. For example, for Bob’s fixed vertex $u_{1}$, the range of $u_{1}+v_{i}$ is $\left[N_{1},N_{1}^{\prime }\right]$; we can then set $u_{2}^{\prime }>N_{1}^{\prime }$, and then all $u_{2}^{\prime }+v_{i}>N_{1}^{\prime }$. The intersection of ranges of $u_{1}+v_{i}$ and $u_{2}^{\prime }+v_{i}$ is empty. By repeating the procedure *m* times, we achieve our goal. In fact, we can do more to relabel the vertices such that the values corresponding to negative edges are odd numbers and the values corresponding to positive edges are even numbers. This is because $v_{i}^{\prime }+u_{j}^{\prime }$ are now different for different edges. If the value is not as what we require, we can add a very large number to make the parity correct. In this way, we see that

(13) $${\left(-1\right)}^{t(x_{A}^{\prime },x_{B}^{\prime })}={\left(-1\right)}^{x_{A}^{\prime }+x_{B}^{\prime }}.$$

Before starting the calculation for a given experimental compatibility graph $G^{e}$, Alice and Bob firstly come together to discuss and fix the procedure in order to conduct the relabeling process. In fact, the easiest way is to relabel the vertices before calculation as $x_{A}^{\prime }$ and $x_{B}^{\prime }$.

With the above preparation, we now present our classical protocol. Alice and Bob, when receiving inputs $\left(x_{A},y_{A}\right)$ and $\left(x_{B},y_{B}\right)$, choose to locally calculate two functions $a(x_{A},\lambda_{A})$ and $b(x_{B},\lambda_{B})$ such that $a\left(x_{A},\lambda_{A}\right)={\left(-1\right)}^{x_{A}^{\prime }}$ and $a\left(x_{A},\lambda_{A}\right)={\left(-1\right)}^{x_{B}^{\prime }}$. Note that, here, $\lambda_{A},\lambda_{B}$ characterize their local classical resources, and they may be classically correlated. Then, Alice and Bob broadcast the results $e_{A}=y_{A}a\left(x_{A},\lambda_{A}\right)$ and $e_{B}=y_{B}b\left(x_{B},\lambda_{B}\right)$, respectively. After receiving the result, they both output it with the answer function

(14) $${\mathrm{Ans}}_{P_{C}}\left(x_{A},y_{A};x_{B},y_{B}\right)=e_{A}e_{B}.$$

The success probability of the protocol is

(15) $$\begin{array}{cc} p_{\mathrm{succ}}\left(P_{C}|C_{bd}=2\right)= & \frac{1}{\left|E(G^{e})\right|}(\sum_{\langle ij\rangle \;\;\mathrm{positive}}p\left(ab=1|v_{i}u_{j}\right)\\ \; & +\sum_{\langle ij\rangle \;\;\mathrm{negative}}p\left(ab=-1|v_{i}u_{j}\right)). \end{array}$$

The protocol can achieve a success probability of $(B_{C}+|E\left(G^{e}\right)|)/2|E\left(G^{e}\right)|$, where $B_{C}$ is the classical bound for Bell inequality. For the 2m-cycle case, it is $p_{\mathrm{succ}}\left(P_{C}|C_{bd}=2\right)=\left(2m-1\right)/2m$, and especially for the well-known CHSH case m=2, $p_{\mathrm{succ}}\left(P_{C}|C_{bd}=2\right)=3/4$.

For the *n*-party case, the protocol works similarly. The main difference is that the experimental compatibility graph is now an *n*-partite hypergraph. By relabeling the vertices, we have

(16) $${\left(-1\right)}^{t(x_{1},\cdots ,x_{n})}={\left(-1\right)}^{x_{1}^{\prime }+\cdots +x_{n}^{\prime }}.$$

After receiving the input bit strings, each party chooses to locally calculate a function $e_{k}=y_{i}a\left(x_{k},\lambda_{k}\right)$ with $a_{k}\left(x_{k},\lambda_{k}\right)={\left(-1\right)}^{x_{k}^{\prime }}$. Finally they broadcast $e_{k}$ simultaneously and output the value

(17) $${\mathrm{Ans}}_{P_{C}}\left(x_{1},y_{1};\cdots ;x_{n},y_{n}\right)=e_{1}\cdots e_{n}.$$

The success probability is similar to Equation (15). The protocol can achieve a success probability of $(B_{C}+|E\left(G^{e}\right)|)/2|E\left(G^{e}\right)|$, where $B_{C}$ is the classical bound for Bell inequality. In this protocol, each party indeed only broadcasts one classical bit of information.

Before we discuss the quantum advantage of the entanglement-assisted protocol, we need to prove that this is in fact the optimal classical protocol.

*Proof of the optimality of the protocol.*—We now show that the above protocol is optimal; i.e., there is no classical protocol reaching a higher success probability. For the two-party case, what we need to show is that when Alice and Bob initially share classical randomness, there is no $C_{bd}=2$ protocol for which Alice and Bob can calculate the function $F_{G^{e}}$ with success probability greater than $(B_{C}+|E\left(G^{e}\right)|)/2|E\left(G^{e}\right)|$. Firstly, we observe that an *n*-bit Boolean function $f(x_{1},\cdots ,x_{n})$ with values ±1 can be decomposed as

(18) $$f\left(x_{1},\cdots ,x_{n}\right)=\sum_{i_{1},\cdots ,i_{n}=0,1}T_{i_{1},\cdots ,i_{n}}x_{1}^{i_{1}}\cdots x_{m}^{i_{n}}.$$

Since $f(x_{1},\cdots ,x_{n})=\pm 1$, we have $|T_{i_{1},\cdots ,i_{n}}|\leq 1$. In fact, the expansion coefficients are given by

(19) $$T_{i_{1},\cdots ,i_{n}}=\frac{1}{2^{n}}\sum_{x_{1},\cdots ,x_{n}=\pm 1}f\left(x_{1},\cdots ,x_{n}\right)x_{1}^{i_{1}}\cdots x_{n}^{i_{n}}.$$

Now, consider the function $F_{G^{e}}\left(x_{A},y_{A};x_{B},y_{B}\right)$; for convenience, we introduce the new variables ${\tilde{x}}_{A}={\left(-1\right)}^{x_{A}^{\prime }}$ and ${\tilde{x}}_{B}={\left(-1\right)}^{x_{B}^{\prime }}$. Using the expansion of Equation (18), the broadcast bits become

(20) $$e_{i}=e_{i}\left({\tilde{x}}_{i},y_{i}\right)=\left(T_{00}+T_{10}{\tilde{x}}_{i}\right)+\left(T_{01}+T_{11}{\tilde{x}}_{i}\right)y_{i}=c_{i}\left({\tilde{x}}_{i}\right)+d_{i}\left({\tilde{x}}_{i}\right)y_{i},$$

where $|c_{i}\left({\tilde{x}}_{i}\right)\left|+\right|d_{i}\left({\tilde{x}}_{i}\right)|=1$ and $|c_{i}\left({\tilde{x}}_{i}\right)\left|,\right|d_{i}\left({\tilde{x}}_{i}\right)|=0,1$, with i=A,B. The inner product of Alice’s answer function with function $F_{G^{e}}$ can be defined as

(21) $$\begin{array}{c} \langle {\mathrm{Ans}}_{A},F_{G^{e}}\rangle =\sum_{\begin{array}{c} x_{A},y_{A},\\ x_{B},y_{B} \end{array}}\frac{\mu (x_{A},x_{B})}{4}{\mathrm{Ans}}_{A}\left(x_{A},y_{A},e_{B}\right)F_{G^{e}}\left(x_{A},y_{A};x_{B},y_{B}\right). \end{array}$$

Here, $\mu (x_{A},x_{B})/4$ is the probability distribution over the inputs. We see that when ${\mathrm{Ans}}_{A}\left(x_{A},y_{A},e_{B}\right)=F_{G^{e}}\left(x_{A},y_{A};x_{B},y_{B}\right)$, they contribute +1 to the above summation; otherwise, they contribute −1. Note the fact that $1=\sum_{x_{A},y_{A},x_{B},y_{B}}\frac{\mu (x_{A},x_{B})}{4}$; the success probability for Alice to output the correct answer can thus be written as $p_{\mathrm{succ}}=\frac{1}{2}\left(1+\langle {\mathrm{Ans}}_{A},F_{G^{e}}\rangle \right)$. Inserting the expression of $F_{G^{e}}$ and the expansion ${\mathrm{Ans}}_{A}\left(x_{A},y_{A},e_{B}\right)={\mathrm{Ans}}_{A}\left({\tilde{x}}_{A},y_{A},e_{B}\right)=\sum_{j_{\tilde{x}}j_{y}j_{e}}T_{j_{\tilde{x}}j_{y}j_{e}}{\tilde{x}}_{A}^{j_{\tilde{x}}}y_{A}^{j_{y}}e_{B}^{j_{e}}$ into it, we obtain

(22) $$p_{\mathrm{succ}}=\frac{\sum_{x_{A},x_{B}}{\left(-1\right)}^{t(x_{A},x_{B})}\left(T_{011}+T_{111}{\tilde{x}}_{A}\right)d_{B}\left({\tilde{x}}_{B}\right)}{\left|E(G^{e})\right|}.$$

From the definition of the expansion coefficients, we have $|T_{011}+T_{111}{\tilde{x}}_{A}|\;\leq \;1$. Using Bell inequality, for arbitrary functions $f(x_{A})$, $g(x_{B})$ with $|f\left(x_{A}\right)|,|g\left(x_{B}\right)|\leq 1$, we have

(23) $$\sum_{x_{A},x_{B}}\gamma_{\langle x_{A}x_{B}\rangle }f\left(x_{A}\right)g\left(x_{B}\right)=\sum_{x_{A},x_{B}}{\left(-1\right)}^{t(x_{A},x_{B})}f\left(x_{A}\right)g\left(x_{B}\right)\leq \frac{B_{C}+\left|E\left(G\right)\right|}{2}.$$

Thus, the success probability must satisfy $p_{\mathrm{succ}}\overset{C}{\leq }\frac{B_{C}+\left|E\left(G^{e}\right)\right|}{2|E(G^{e})|}$. Since the protocol we gave before reaches the bound, it is the optimal classical protocol. Similarly for Bob, we can define $\langle {\mathrm{Ans}}_{B},F_{G^{e}}\rangle$. From symmetry of the problem expression, the same result holds for Bob. For the *n*-party case, the proof is completely the same.

The proof here is similar in character to that in Ref. [10]. Another way to prove optimality is to use the traditional communication complexity theoretic approach, for which we first prove a lower bound of the deterministic protocol. Then, using a famous theorem [29]—which states that the communication complexity $R_{\epsilon }\left(f\right)$ of the randomized protocol for computing the function *f* with error ϵ has a relationship with the communication complexity $D_{\epsilon }\left(f|\mu \right)$ of the deterministic protocol for computing the function *f* with error ϵ, for which inputs are distributed with μ as $R_{\epsilon }\left(f\right)=\max_{\mu }D_{\epsilon }\left(f|\mu \right)$—the lower bound of the deterministic protocol can be proved by assuming a protocol tree with a depth of 2 (for the two-party case) and discussing the partitions of the inputs by different nodes of the protocol tree.

### 4.2. Entanglement-Assisted Protocol

The quantum protocol works as follows: We take the two-party case as an example. Alice and Bob pre-share an entangled quantum state ${|\psi \rangle }_{AB}$, upon which Alice and Bob can choose ±1-valued observables $A_{1},\cdots ,A_{m}$ and $B_{1},\cdots ,B_{n}$ and obtain a violated value of Bell inequality corresponding to the experimental compatibility graph $G^{e}$. Now, if Alice and Bob receive input values $x_{A}=v_{i}$ and $x_{B}=u_{j}$, they can measure the corresponding observables $A_{i}$ and $B_{j}$ and output $a_{A}=a_{i}$ and $b_{B}=b_{j}$. Then, Alice and Bob broadcast the classical bits $e_{A}=y_{A}a_{A}$ and $e_{B}=y_{B}b_{B}$, respectively. After receiving the communicated bits, Alice and Bob both give their answers as ${\mathrm{Ans}}_{A}={\mathrm{Ans}}_{B}=e_{A}e_{B}$. The success probability is still Equation (15). We see that it can exceed the bound of classical protocol, thus exhibiting the quantum advantage.

For further clarification, let us first take $G_{2m-cycle}^{e}$ as an example (see Example 1). Suppose that Alice and Bob pre-share the singlet state $|\psi^{-}\rangle =\frac{1}{\sqrt{2}}(|01\rangle -|10\rangle ).$ The observables for Alice are $A_{i}=m_{i}\cdot \sigma$, where

$$m_{i}=\left(\cos\frac{(2i-1)\pi }{2m},0,\sin\frac{(2i-1)\pi }{2m}\right),\;\;i=1,\cdots ,m,$$

and for Bob, they are $B_{j}=n_{j}\cdot \sigma$, where

$$n_{j}=\left(\cos\frac{j\pi }{m},0,\sin\frac{j\pi }{m}\right),\;\;j=1,\cdots ,m.$$

With these measurements, Alice and Bob can achieve a success probability $p_{\mathrm{succ}}=\frac{\cos\pi /2m+1}{2}$, which corresponds to the Tsirelson bound of the 2m-cycle Bell inequality. We note that the success probability is a monotone increasing function, and when m→∞, it tends to 1.

Another example is $G_{2|3}^{e}$, as illustrated in Example 2. Alice and Bob still pre-share the singlet state, and Alice chooses to measure $A_{1}=\sigma_{x}$ and $A_{2}=\sigma_{z}$, while Bob chooses to measure $B_{j}=n_{j}\cdot \sigma$ with

$$\begin{array}{cc} \; & n_{1}=\left(\cos\frac{\pi }{4},0,\cos\frac{\pi }{4}\right),\\ \; & n_{1}=\left(\cos\frac{3\pi }{4},0,\cos\frac{3\pi }{4}\right),\\ \; & n_{3}=\left(\cos\left(\frac{\pi }{4}+\theta \right),0,\cos\left(\frac{3\pi }{4}+\theta \right)\right),\theta \ll 1. \end{array}$$

The optimal classical protocol can achieve a success probability of 5/6. Here, the quantum protocol can almost reach the success probability of $(3\sqrt{2}+6)/12$ for sufficiently small θ, which is greater than the success probability for the optimal classical protocol; thus, it exhibits a quantum advantage. Note that the above problem is closely related to the problem of simulation of nonlocal correlation via classical communication [33]. Our result here matches well with previous results that suggested that by two bits of classical communication, Bell nonlocal measurement statistics can be simulated.

### 4.3. Popescu–Rohrlich Box Protocol

Let us now consider a non-signaling world, which is beyond quantum mechanics. Suppose that Alice and Bob pre-share a black box such that for the positive edge 〈ij〉 of the experimental compatibility graph $G^{e}$, the probability distribution of outputs for measurements $A_{i},B_{j}$ is

(24) $$p\left(a_{i},b_{j}|A_{i},B_{j}\right)=\left\{\begin{array}{c} 1/2,\;a_{i}b_{j}=1,\\ 0,\;a_{i}b_{j}=-1. \end{array}\right.$$

Additionally, for negative edges, the distribution is

(25) $$p\left(a_{i},b_{j}|A_{i},B_{j}\right)=\left\{\begin{array}{c} 0,\;a_{i}b_{j}=1,\\ 1/2,\;a_{i}b_{j}=-1. \end{array}\right.$$

This kind of black box is known as a Popescu–Rohrlich box [18] or a perfect nonlocal box. It can be easily confirmed whether the box satisfies the non-signaling principle.

With the help of the Popescu–Rohrlich box, we can reach a success probability of $p_{\mathrm{succ}}=1$. The protocol works in a similar manner to that of the entanglement-assisted protocol. After receiving the inputs $x_{A}^{\prime }=v_{i}$ and $x_{B}^{\prime }=u_{j}$, Alice and Bob choose to measure $A_{i}$ and $B_{j}$ jointly and output $a_{i}$ and $b_{j}$ with a probability of $p(a_{i},b_{j}|A_{i},B_{j})$. After many runs of the experiment, Alice and Bob check their success probability. It is obvious from Equation (15) that for the Popescu–Rohrlich box, the success probability is $p_{\mathrm{succ}}=1$. This matches well with the result in Refs. [34,35], which states that using a perfect nonlocal box can make CCP trivial for arbitrary Boolean function.

## 5. Conclusions and Discussions

Identifying the bound of classical theory and quantum theory is of great importance for understanding the nature of our universe. In this work, we attempt to gain a better understanding of the problem from a communication complexity theoretic perspective. By restricting the classical communications, two parties can calculate a given function with different success probabilities; this shows that the strength of quantum correlations is much stronger than that of the classical one. These results shed new light on the bound between classical and quantum worlds. From a practical point of view, our result provides a method to construct a CC function from an arbitrary given experimental compatibility graph or hypergraph. When the graph is a bipartite graph, it gives a two-party CC function, and when the graph is multipartite, it gives a multi-party CC function. Our construction may have potential applications in practical CC problems where quantum advantages from Bell nonlocality need to be extracted.

## Figures and Tables

**Table 1:** Value of $F_{G_{6-cycle}^{e}}$; columns are indexed by $\left(x_{A},y_{A}\right)$ and rows are indexed by $\left(x_{B},y_{B}\right)$.

	(1, +1)	(1, −1)	(2, +1)	(2, −1)	(3, +1)	(3, −1)
(1, +1)	1	−1	1	−1	−	−
(1, −1)	−1	1	−1	1	−	−
(2, +1)	−	−	1	−1	1	−1
(2, −1)	−	−	−1	1	−1	1
(3, +1)	1	−1	−	−	−1	1
(3, −1)	−1	1	−	−	1	−1

## Data Availability

Not applicable.
